# Postoperative Outcomes After Emergency Surgery in COVID-19 Patients: An Ambispective Matched Cohort Study

**DOI:** 10.7759/cureus.55845

**Published:** 2024-03-09

**Authors:** Sunaina T Karna, Zainab Ahmad, Pooja Thaware, Saurabh Trivedi, Revadi Gouroumourty, Pooja Singh, Vaishali Waindeskar, Jai Prakash Sharma, Ashutosh Kaushal, Saurabh Saigal

**Affiliations:** 1 Anesthesiology, All India Institute of Medical Sciences, Bhopal, Bhopal, IND; 2 Anesthesiology, Chirayu Medical College and Hospital, Bhopal, IND; 3 Community & Family Medicine, All India Institute of Medical Sciences, Bhopal, Bhopal, IND

**Keywords:** sars-cov-2 (severe acute respiratory syndrome coronavirus -2), mortality, morbidity, emergency surgery, covid-19

## Abstract

Purpose

There is limited data from the Indian subcontinent regarding the surgical outcomes of coronavirus disease (COVID-19) patients. In this observational study, we aimed to evaluate the postoperative outcomes after emergency surgery in COVID-19 patients compared to concurrent age and gender-matched controls. We also sought to analyze the possible predictors of postoperative mortality in COVID-19 patients.

Methods

This matched cohort study was conducted in a tertiary care teaching hospital in central India, between 1st July 2021 and 30th June 2022. COVID-19-positive patients undergoing emergency surgery under anesthesia were recruited as cases. Age and gender-matched COVID-19-negative patients undergoing a similar nature of surgery in the same period served as concurrent controls. The cases and controls were compared for the 30-day mortality and perioperative complications.

Results

The COVID-19-positive surgical cohort had a 12.3 times greater 30-day postoperative overall mortality risk as compared to a matched cohort of patients with a negative COVID-19 test. A positive COVID-19 status was associated with more postoperative complications of acute respiratory distress syndrome (ARDS), sepsis, shock, and persistent hyperglycemia. On analysis of predictors of mortality, the presence of preoperative dyspnea, ARDS, American Society of Anesthesiologists Physical Status (ASA-PS) Class IIIE/IVE, increase in sequential organ failure assessment (SOFA) score, Quick SOFA>1, higher creatinine, bilirubin, and lower albumin were observed to be associated with increased mortality.

Conclusions

Severe acute respiratory syndrome coronavirus 2 (SARS-CoV-2) infection in patients undergoing emergency surgery is significantly associated with higher postoperative complications and increased 30-day postoperative mortality.

## Introduction

The novel coronavirus disease (COVID-19) pandemic, which started in December 2019, led to significant upheavals in the healthcare system. Most global studies on surgical patients showed worse perioperative outcomes in patients with a positive severe acute respiratory syndrome coronavirus 2 (SARS-CoV-2) test [1-6]. The few Indian studies that are available also report high morbidity and mortality in COVID-19 patients after surgery but are retrospective in nature [7,8]. Understanding the impact of COVID-19 infection on postoperative morbidity and mortality in surgical patients is vital for evidence-based preoperative risk stratification, shared decision-making, balancing risks with benefits, monitoring trends in postoperative outcomes, and planning surgical services for future pandemics. 

This ambispective observational matched cohort study aimed to estimate and compare postoperative outcomes, specifically, 30-day postoperative morbidity and mortality in COVID-19-positive patients undergoing emergency surgery with concurrent controls. We also aimed to assess the predictors of mortality in COVID-19 patients undergoing emergency surgery.

## Materials and methods

Materials and methods

This ambispective observational matched cohort study was conducted in a tertiary care teaching institute in central India, a dedicated 500-bed referral center for COVID-19 patients, during the second wave of this pandemic (March to November 2020 and March to May 2021). The study was approved by the Institutional Humans Ethical Committee (IHEC LOP/2021/ IM0375) and registered with the Clinical Trials Registry of India (CTRI/2021/12/038975). All study participants gave informed consent to use their anonymized data for scientific purposes. Written informed consent was taken before surgery in prospectively enrolled study participants. In patients enrolled retrospectively, the consent was taken telephonically. This study complies with the Strengthening the Reporting of Observational Studies in Epidemiology (STROBE) guidelines.

Objective

The primary objective of this study was to estimate the 30-day mortality rate in COVID-19 patients undergoing emergency surgery compared to age and sex-matched concurrent controls without COVID-19 undergoing similar types of emergency surgery. The secondary objectives were to estimate the postoperative morbidity rate, the need for interventions like mechanical ventilation, vasoactive drugs, dialysis, re-operation, and length of hospital stay in both cohorts, and evaluate the predictors of 30-day mortality in COVID-19 patients.

Study population

All consecutive patients who underwent an emergency surgical procedure under anesthesia between 1st July 2021 and 30th June 2022 and had a positive test result for SARS-CoV-2 infection were included in the COVID-19 cohort. SARS-CoV-2 infection was diagnosed based on a positive reverse transcriptase polymerase chain reaction (RT-PCR) of a paired nasopharyngeal and oropharyngeal swab one month preoperatively or within one week after surgery. We excluded patients who underwent minor surgeries under local anesthesia. Study participants belonging to the COVID-19 cohort were recruited retrospectively as well as prospectively. The study participants in the COVID-19 cohort were further matched prospectively based on age group and sex to COVID-19 negative concurrent controls undergoing a similar type of surgery, in a 1:2 ratio during the same study period.

Data collection 

The following data was collected on a computer-based database by an independent study investigator. Patient age, sex, body mass index (BMI), clinical symptoms suggestive of COVID-19, presence of comorbidities, vital signs, organ-specific complications at admission, the timing of the SARS-CoV-2 test, and other preoperative laboratory tests were recorded. Risk indices, i.e., the American Society of Anesthesiologists-Physical Status (ASA-PS), Sequential Organ Failure Assessment (SOFA) score, and quick-SOFA (q-SOFA) score were calculated. The duration and nature of surgery were noted along with perioperative anesthetic details, including the type of anesthesia, airway, and ventilator management.

A second team of investigators did weekly follow-up visits till one month after surgery or death in cases recruited prospectively. In cases recruited retrospectively, computerized electronic daily records and laboratory investigations were evaluated besides the daily clinical progress sheets from the medical records department. Complications were recorded according to the Clavien Dindo scale [9,10]. The presence of renal acute kidney injury (AKI), cardiac (arrhythmias, poor contractility), neurological, respiratory acute respiratory distress syndrome (ARDS), thrombotic, acute liver injury, shock, sepsis, and persistent hyperglycemia were recorded. The length of invasive mechanical ventilation, intensive care, and hospital stay, as well as mortality at seven days and 30 days after surgery, were also documented.

Statistical analysis

Data was entered, cleaned, and coded using Microsoft Excel software version 2010. R Core Team version 4.2.1 (2020, Austria) was used for all statistical tests. Numerical descriptive data has been summarized as mean standard deviation (SD) or median + interquartile range (IQR) based on the distribution, and categorical variables were summarized as percentages. The χ2 or Fisher exact test was used to evaluate the categorical association between COVID-19 positivity and the occurrence of morbidity or mortality. The unpaired T-test or Mann-Whitney U test was performed to evaluate whether a difference in length of stay existed based on COVID-19 status. The incidence risk ratio was evaluated to identify the risk of postoperative complications among the COVID-19 patients compared to the controls. The proportionate mortality rate was estimated for both cohorts separately. Cox proportional hazard estimation was used for the analysis of the survival times. Univariate Cox proportional hazards regression analysis was done to find predictors of 30-day mortality from the time of symptoms to surgery reported as coefficient hazard ratio and 95% confidence interval. Kaplan Meier survival analysis was used to determine the probability of survival past the given days after surgery. 

Sample size calculation

At the time of the commencement of the study, since Indian studies were not available, we calculated the sample size from a large international multicentric study. In this study, the mortality rate at one month in surgical patients with COVID-19 was observed to be 19.5% compared to 2.44% in matched controls without COVID-19 [11]. considered an alpha error of 5%, beta error of 20%, power of study at 80%, and confidence level at 95%. The sample size was calculated to be 43 patients in the COVID-19 cohort. To match in a 1:2 ratio, we planned to recruit 86 patients in the control group, with a total sample size of 129 patients. A p-value < 0.05 was considered significant.

## Results

Description of the cohort

**Figure 1:**
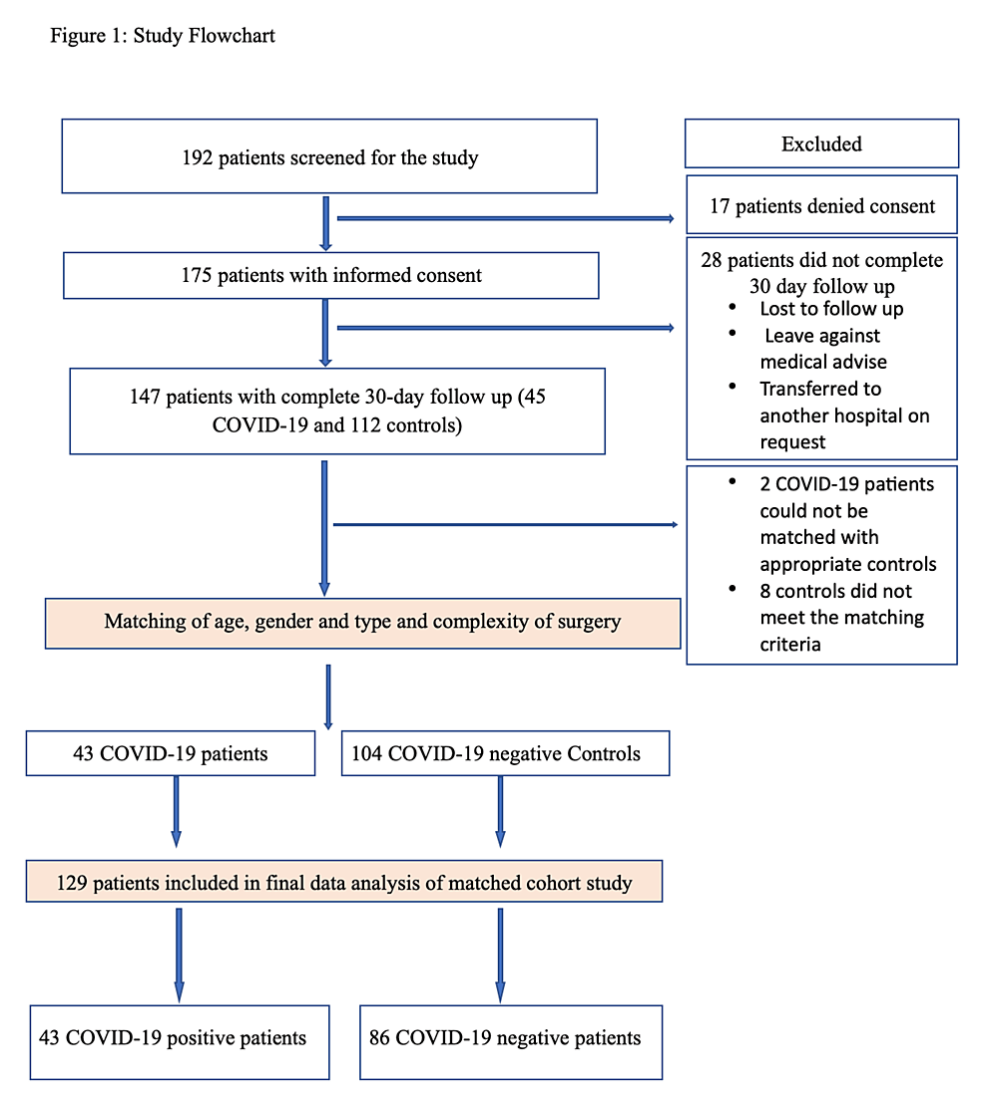
Study flowchart

A total of 192 patients undergoing emergency surgery during the pandemic were screened for inclusion in the study period. The participant flowchart is depicted in Figure 1. A total of 43 COVID-19-positive patients and 86 controls after age, sex, and surgical type/complexity matching with complete 30-day follow-up were included in the final analysis.

Table 1 depicts the baseline characteristics of the study participants. The most significant prevalent symptom observed in the COVID-19 cohort was fever, followed by cough and dyspnea. Significantly more COVID-19 patients were co-morbid with two or more comorbidities versus controls (48% vs 27%). Preoperative intensive care was needed in eight (19%) of patients in the COVID-19 cohort versus six (7%) in controls, this difference was not statistically significant (p-value 0.069). SOFA score was worse in COVID-19 patients, and qSOFA >1 was also commoner in cases vs controls (25.3% vs 8.1%). The surgical procedures performed were 39 (32%) otorhinolaryngology, 18 (15 %)abdominal, 57 (46%) obstetric, six (4.9%) neurosurgical, and three (3.4%) orthopedic. 

**Table 1:** Baseline descriptive characteristics of the matched cohort (n=129) BMI: body mass index; SOFA: sequential organ failure assessment; qSOFA: quick SOFA; GIT: gastrointestinal tract.

Characteristic	Overall n=129	COVID-19 positive n=43	COVID-19 negative n=86	p-value
Gender				0.8
Male	49 (38%)	17 (40%)	32 (37%)	
Female	80 (62%)	26 (60%)	54 (63%)	
Age group				>0.9
Less than 40 years	66 (51%)	22 (51%)	44 (51%)	
40-59 years	33 (26%)	11 (26%)	22 (26%)	
60-79 years	27 (21%)	9 (21%)	18 (21%)	
More than 80 years	3 (2.3%)	1 (2.3%)	2 (2.3%)	
BMI classification				0.5
Less than 18.5	2 (1.6%)	0 (0%)	2 (2.3%)	
18.5-24.9	77 (60%)	23 (53%)	54 (63%)	
25-29.9	43 (33%)	18 (42%)	25 (29%)	
30-34.9	7 (5.4%)	2 (4.7%)	5 (5.8%)	
More than 40	0 (0%)	0 (0%)	0 (0%)	
Symptoms	68 (53%)	31 (72%)	37 (43%)	0.002
Fever	32 (25%)	18 (42%)	14 (16%)	0.002
Cough	17 (13%)	13 (30%)	4 (4.7%)	<0.001
Dyspnea	16 (12%)	9 (21%)	7 (8.1%)	0.038
Comorbidities				0.07
Single	48 (67%)	12 (52%)	36 (73%)	
Two or more comorbidities	24 (33%)	11(48%)	13 (27%)	
Scores				
SOFA	1.2 (2.3)	2.0 (2.6)	1.0 (2.0)	0.002
qSOFA				0.009
Grade 0	111(86%)	32 (74%)	79 (92%)	
Grade 1	15 (12%)	10 (23%)	5 (5.8%)	
Grade 2	3 (2.3%)	1 (2.3%)	2 (2.3%)	
Surgical specialty				>0.9
General/GIT	18 (15%)	6 (13.9%)	12 (13.9%)	
Orthopedic	3 (2.4%)	1 (2.3%)	2 (2.3%)	
Neurosurgery	6 (4.9%)	2 (4.7%)	4 (4.7%)	
Obstetric	57 (46%)	19 (44%)	38 (44%)	
Oto-rhino-laryngology	39 (32%)	13 (30%)	26 (30%)	

Surgical treatment and anesthesia in patients with COVID-19

Out of 43 cases, 39 (91%) were diagnosed as COVID-19 positive preoperatively with a median (interquartile range) time of 1.0 (1.0, 3.0) days from diagnosis to surgery. Only four (9.3%) patients were diagnosed postoperatively. The details of the surgical procedures performed are mentioned in Appendix 1. The type and complexity of surgery were comparable between the two cohorts. Procedures were performed under general anesthesia for 21 patients. Subarachnoid and brachial plexus block were administered in 21 and one patient, respectively. Overall, the intraoperative complications were similar between both groups. In patients on controlled ventilation after administration of general anesthesia, reduced lung compliance was seen in 9.3% of patients in the COVID-19 cohort compared to 2.3% in controls. However, this difference was not statistically significant (p-value 0.095).

Postoperative Outcomes

The COVID-19 cohort experienced worse postoperative outcomes than the controls. Postoperative complications were present in 33% versus 16% of patients with and without COVID-19, respectively (p-value 0.038) as seen in Table 2. 

**Table 2:** Peri-operative complications and post-operative 30-day outcomes in COVID-19 cohort versus controls ARDS: acute respiratory distress syndrome, AKI: acute kidney injury.

Characteristic	Total n= 129	COVID-19 positive n=43	COVID-19 negative n=86	p-value
Intra-operative complications				
Adverse events	16 (12%)	7 (16%)	9 (10%)	0.3
Hypotension	14 (11%)	7 (16%)	7 (8.1%)	0.2
Reduced respiratory compliance	6 (4.7%)	4 (9.3%)	2 (2.3%)	0.095
Acute kidney injury (AKI)	4 (3.1%)	3 (7%)	1 (1.2%)	0.11
Postoperative complications				
Present	28 (22%)	14 (33%)	14 (16%)	0.038
Post-operative ARDS	21 (16.3%)	9 (20.9%)	8 (9.3%)	<0.001
Severity of ARDS				
Mild	13 (10%)	5 (12%)	8 (9.3%)	<0.001
Moderate	4 (3.1%)	4 (9.3%)	0	
Severe	4 (3.1%)	4 (9.3%)	0	
Sepsis	16 (12%)	11 (26%)	5 (5.8%)	0.001
Shock	14 (11%)	11 (26%)	3 (3.5%)	0.001
Persistent hyperglycemia	5 (3.9%)	5 (12%)	0	0.004
Arrhythmias	3 (2.3%)	2 (4.7%)	1 (1.2%)	0.3
Neurological complications	7 (5.4%)	3 (7.0%)	4 (4.7%)	0.7
Thrombotic complications	2 (1.6%)	2 (4.7%)	0	0.11
Acute liver injury	20 (16%)	7 (16%)	13 (15%)	0.9
Severity of AKI				
Absent	109 (84%)	34 (79%)	75 (87%)	0.2
Mild	16 (12%)	6 (14%)	10 (12%)	
Moderate	2 (1.6%)	2 (4.7%)	0	
Severe	2 (1.6%)	1 (1.2%)	1 (1.2%)	
Clavien Dindo Score				
No morbidity	50 (39%)	0 (0%)	50 (58%)	<0.001
Class 1	43 (33%)	20 (47%)	23 (27%)	
Class 2	9 (7.0%)	8 (19%)	1 (1.2%)	
Class 3A	9 (7.0%)	0 (0%)	9 (10%)	
Class 3B	4 (3.1%)	2 (4.7%)	2 (2.3%)	
Class 4A	1 (0.8%)	1 (2.3%)	0 (0%)	
Class 4B	2 (1.6%)	2 (4.7%)	0 (0%)	
Class 5	11 (8.5%)	10 (23%)	1 (1.2%)	
Interventions				
Intensive care n=37	4.0 (2.2, 11.0) n=30	3.5 (2.2,11.0), n=14	4.0 (2.8,8.2) n=16	0.3
Mechanical ventilation n=28	3.0 (1.0, 6.0) n=21	3.5 (3.0, 7.5) n=10	3.0 (1.0, 5.5) n=11	0.5
Length of hospital stay	11.0 (7.0, 25.0)	12.0(7.0, 28.5)	9.5(6.0, 24.0)	0.3
Postoperative Mortality				
7-day mortality	12 (9.3%)	6 (14%)	6 (7.0%)	0.2
30-day mortality	11 (8.5%)	10 (23%)	1 (1.2%)	<0.001

Postoperative ARDS was more frequent in the COVID-19 cohort (20.9% vs. 9.3%, p-value < 0.001), with higher severity of ARDS seen only in the COVID-19 cohort (12% mild, 9.3% moderate, and 9.3% severe). In the control group, postoperative ARDS was seen in only 9.3% of patients, which was of mild severity. Postoperative complications of sepsis, shock, and persistent hyperglycemia were more frequent in the COVID-19 cohort, as seen in Table 2. Postoperative morbidity assessed by Clavien Dindo score (CDS) was absent in 52% of patients belonging to the control group. On the other hand, some form of postoperative morbidity was present in every patient who was COVID-19 positive, with the presence of CDS higher than 3B in 14 (32.5%) patients. 

Median length of mechanical ventilation, intensive care, and hospital stay were similar in patients with and without COVID-19 infection. A statistically significant association of a positive COVID-19 status was also observed with 30-day mortality (Table 2). 

**Figure 2:**
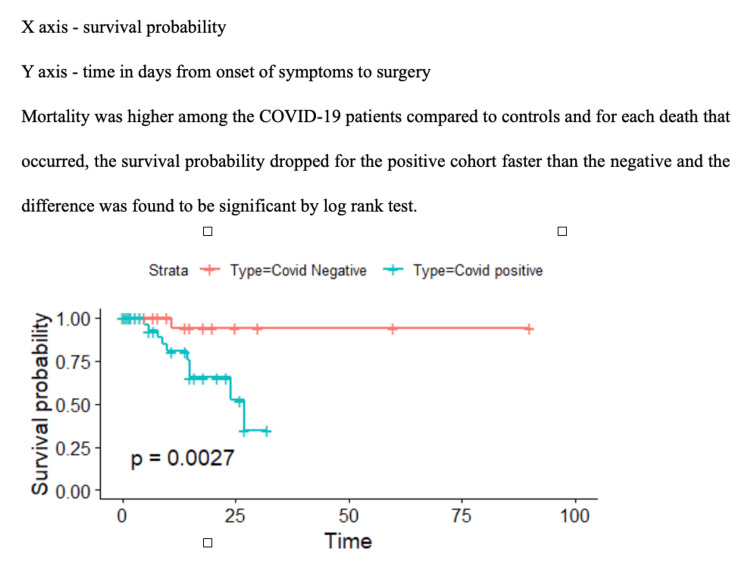
Kaplan Meir curve showing survival probability in COVID-19 patients and controls against time from onset of symptom to surgery X axis: survival probability; Y axis: time in days from onset of symptoms to surgery; Mortality was higher among the COVID-19 patients compared to controls and for each death that occurred, the survival probability dropped for the positive cohort faster than the negative and the difference was found to be significant by log-rank test.

The 30-day postoperative mortality was high in COVID-19 patients versus matched controls (23% vs. 1.2%, p-value < 0.001). Using the Cox regression model along the time from onset of symptom to surgery, there were 11 deaths at the end of 30 days among all included patients. Of these, 10 were in cases, and one was in control. The cases had a 12.3 times higher risk of 30-day mortality (95% CI 1.55,97.2, p-value 0.018) than the controls. The first death among the cases appeared on the fifth day from the onset of symptom to surgery, and the survival probability was 96% at that point of time with 95% CI (0.89-1.00), as shown in Figure 2.

On univariate Cox proportional hazards regression analysis (Table 3), the following factors were significant predictors of 30-day postoperative mortality - ASA grade III E/ IVE status [HR 36.6 (95% CI 4.66, 288), p-value < 0.001], a COVID-19 positive status [HR 12.3 (95% CI 1.55, 97.2), p-value < 0.018], postoperative AKI of any severity [HR 23.3 (95% CI 2.87, 190), p-value 0.003].

**Table 3:** Univariate Cox proportional hazards regression for prediction of mortality from the time of symptom to surgery ASA: American Society of Anesthesiologists; ARDS: acute respiratory distress syndrome; HR: hazard ratio; CI: confidence interval.

Characteristic	HR	95% CI	p-value
Age group			
40-59 years	0.14	0.01, 1.56	0.11
60-79 years	1.71	0.35, 8.42	0.51
> 80 years	2.54	0.23, 28.6	0.45
Gender	1.69	0.52, 5.57	0.39
>2 Comorbidties	2.14	0.21, 21.4	0.52
ASA IIIE/ IVE	36.6	4.66, 288	<0.001
Diabetes Mellitus	2.25	0.64, 7.84	0.20
Peripheral vascular disease	0.36	0.04, 2.93	0.34
Malignancy	0.58	0.07, 4.59	0.61
Preoperative			
Absence of dyspnea	0.18	0.06, 0.60	0.005
Absence of Fever	0.53	0.15, 1.82	0.31
Absence of cough	0.60	0.15, 2.37	0.47
COVID-19 infection	12.3	1.55, 97.2	0.018
Absence of ARDS	0.11	0.02, 0.50	0.004
Laboratory investigations			
Serum creatinine	2.42	1.04, 5.63	0.040
Serum bilirubin	3.55	1.53, 8.23	0.003
Serum albumin	0.21	0.07, 0.64	0.006

There was a decreased risk of mortality in the absence of preoperative dyspnea [HR 0.18 (95% CI 0.06, 0.6), p-value 0.005], preoperative ARDS [HR 0.11 (95% CI 0.02, 0.50), p-value 0.004] and postoperative liver injury [HR 0.15 (95% CI 0.05, 0.51), p-value 0.002]. The risk of mortality increased with an increase in SOFA [HR 1.82 (95% CI 1.43, 2.32), p-value < 0.001]. Quick SOFA scores of 1 and 2 were associated with a 12.1 and 45.7 times increase in mortality risk, respectively. Every one-unit increase in creatinine and bilirubin raised mortality risk by 1.42 (p-value 0.04) and 2.55 (p-value 0.003) times, respectively. Each unit increase in albumin was associated with a reduced risk of death [HR 0.21 (95% CI 0.07, 0.64), p-value 0.006]. 

## Discussion

The main focus of this matched cohort study conducted in a tertiary care institute in central India was to investigate the mortality and morbidity rates between patients with and without a positive SARS-CoV-2 test undergoing emergency (immediate) and urgent (needed within 24-48 hours) during the COVID-19 pandemic. 

In the early phase of the pandemic, a case series of 34 COVID-19 surgical patients from China reported poor outcomes with the incidence of postoperative pneumonia, ARDS, and shock in 100, 32, and 29% of patients, respectively, with a perioperative mortality rate of 21% [12]. A mortality rate of 19.5% was observed in 41 COVID-19-positive surgical patients treated by different subspecialties (neurosurgery, orthopedics, general, thoracic, and vascular surgery) in a single hospital in Lombardi, Italy [13]. Later, the COVID-SURG collaborative reported an overall postoperative mortality rate of 23.8% in COVID-19 patients. On subgrouping according to the nature of surgery, higher mortality rates were observed after major (26.9%) or emergency (25.6%) surgeries [6]. In 2021, Beatty et al., in a multicentric observational PREDICT study conducted by 55 teams across 18 countries, observed an 18.9% mortality rate (OR 5.12, CI 2.06-12.72) in surgical interventions in 1342 patients [14]. Though collaborative studies like COVID-SURG and PREDICT have been very successful in providing rapid information in perioperative research, they cannot replace smaller single-center or multicenter studies with more detailed prospective data collection, as was stressed by a recent editorial [15]. Further, the suggestions of COVIDSurg and GlobalSurg Collaborative of waiting till seven weeks from COVID-19 infection or resolving symptoms before undertaking surgery to decrease mortality is primarily applicable for elective surgery. It may not be possible to defer emergency or urgent surgery for this suggested period. Recent studies have also observed an increased risk of mortality after emergency surgery [HR 6.35, (3.39,11.89), p-value < 0.001] in COVID-19 patients as compared to elective surgery [16]. 

Most data on postoperative mortality in surgical patients with COVID-19 infection is from China, Europe, the United States of America, and the United Kingdom. Further, the studies are limited to different surgical subspecialties [14,17]. In 2020, in a retrospective analysis of surgical outcomes of COVID-19 patients admitted for surgery in a tertiary care hospital in North India, 13 out of 25 patients who underwent surgery died. Mortality was significantly higher in patients admitted for emergency surgery (52.9%) versus elective surgery (10.5%) [18]. Regarding studies in matched cohorts with and without COVID-19 infection, these were limited in number, with no availability of data from India. We observed a 23% postoperative 30-day mortality rate in our COVID-19 patient cohort from central India. Our reported mortality rate in COVID-19 patients is higher than that reported in the PREDICT study, presumably because we have included only emergent or urgent surgeries, which have higher mortality than elective ones [14]. 

In contrast to our findings, Jonker et al. observed no statistical difference between elective and emergency surgery in the 26 non-survivors in their matched cohort study in patients with and without COVID-19 disease [5]. Hence, our matched cohort study may be able to provide evidence for prognostication regarding postoperative outcomes in such emergency cases. In a similar study from Russia with 48 COVID-19 patients with urgent and emergent surgical diseases (malignancies, trauma, acute abdomen, etc.), a significantly increased in-hospital mortality by 8 times (31.3%) and postoperative mortality by 6.5 times (33.3%) were observed compared to the pre-pandemic period [19].

We observed that though there was no statistically significant difference in respiratory compliance during surgery in cases and controls, the most frequent postoperative complication was ARDS, which led to postoperative respiratory insufficiency. It is interesting to note that postoperative ARDS was not only significantly higher (20.9%) but also more severe in our COVID-19-positive cohort. As the lung is the main target organ of the virus, our findings are consistent with that reported by Chen et al., who showed that 17% of patients developed ARDS, of which 11% deteriorated [20]. The seven-day postoperative period was chosen to reflect the mean incubation period of SARS-CoV-2 infection and the assumption that a positive PCR test within this period likely reflected preoperative viral exposure [21]. Further, sepsis and shock were seen more frequently in our COVID-19-positive cohort. Mechanical ventilation, anesthesia, or tissue damage caused by surgery may each provoke a pro-inflammatory cytokine and immunosuppressive response, which may worsen the presentation of the disease. The persistent hyperglycemia seen after surgery in our COVID-19 cohort is possible because of the use of steroids as a treatment regimen in COVID-19 ARDS or for refractory hypotension in septic shock. 

The increased mortality in COVID-19 patients is not surprising. Previous literature has attributed it to a synergistic effect of SARS-CoV-2 infection and surgery as the patient’s immune function is a significant determinant of disease severity. Surgical stress may be responsible for impairment of immune function and induction of systemic inflammatory response [17,22,23]. Still, it is uncertain as to why mortality after COVID-19 surgical cohort is higher in India as compared to matched cohorts from other countries. Previously, during the COVID-19 pandemic, the reported deaths per 1000 residents were 1.6-2.1 in developed countries, while it was reported relatively high at 5.2 in Chennai [24]. However, whether the socioeconomic disparity contributed to excess mortality in our study cohort or it was because of greater severity of presenting surgical pathologies, possibly caused by delayed presentation to the hospital, weaker immune response, or synergistic action of SARS-CoV-2 and surgery is not clear [25]. Further with the progression of the pandemic, natural selection and evolution selection of the genomic variants of SARS-CoV-2) associated with higher transmission, severe disease, re-infection, and immune escape may be a reason for higher mortality in our patients [26]. The findings of the current study have direct implications for assessing the burden of COVID-19 on perioperative outcomes. 

On analysis of predictors of mortality in the matched cohort, the presence of ASA IIIE/IVE, positive SARS-CoV-2 test, Quick SOFA>1, increase in SOFA, higher creatinine, bilirubin, liver enzymes, and presence of postoperative AKI, acute liver injury were all observed to be associated with increased mortality. We observed a protective effect of the absence of preoperative dyspnea and ARDS on mortality, which was also seen in previous studies [17]. 

The strength of this study is that the cohort of COVID-19 patients was closely matched with controls with respect to age, gender, and type and complexity of surgery. With the advantage of a single center, tight matching was possible so that the confounding factors were reduced. Further, the controls are concurrent and not historical as was used in many previous studies, thus ensuring similar logistic and clinical conditions for all study participants. This is the first cohort study to use a well-matched control group to provide good, evidence-based support for this clinical observation of increased mortality associated with COVID-19 infection. Since preoperative COVID-19 testing was mandatory, the high testing rates led to a higher COVID-19 prevalence in our study. The underestimation of COVID-19 has been identified in previous studies on surgical patients due to low testing rates [6,27].

This study has many limitations. First, the study is a single center with a relatively small sample size, which does not allow detailed subpopulation analysis, highlighting the limited number of surgeries done during the pandemic. However, this study focuses on the association of a positive SARS-CoV-2 test with postoperative outcomes. Though our study may shed light on the dynamic response to the locoregional burden of emergency surgery in central India, it may be different from practices in other health systems. Second, the follow-up period is limited to one month, and only early outcomes were investigated. The data of different surgical specialties is included. Future studies with larger cohorts or systematic meta-analyses of published matched cohort studies from multiple centers are needed to determine the effects of COVID-19 in different surgical subspecialties and clinical scenarios. 

## Conclusions

In conclusion, this matched cohort study from central India highlights that a COVID-19-positive status within 30 days before or one week after emergency surgery is associated with an increase in the 30-day overall postoperative mortality rate and complications. Further, an increased risk of mortality is present if the ASA grade is IIIE/IVE, Quick SOFA>1, there is an increase in SOFA, higher creatinine, bilirubin, and the presence of postoperative AKI. Absence or preoperative ARDS, dyspnea, and postoperative liver injury also protect from mortality. 

## Appendices

Appendix 1 

**Table 4:** Details of surgical procedures performed in the matched cohort ORIF: open reduction and internal fixation; LSCS: lower segment cesarean; FESS: functional endoscopic sinus surgery.

Procedure	Matched cohort (N=129)
Neurosurgery	
Decompressive craniotomy for Intracranial bleed with mass effect	6 (4.65%)
Abdominal surgery	
Resection and anastomosis small bowel- Bowel gangrene/obstruction	6 (3.87%)
Exploratory laparotomy with Omental patch	3 (3.1%)
Ileal resection with ileostomy for perforation	6 (4.65%)
Pancreatic necrosectomy for pancreatitis	3 (2.3%)
General Surgery	
Below elbow amputation for gangrene	3 (2.3%)
Toe amputation for toe gangrene	3 (2.3%)
Orthopedic surgery	
ORIF Femur fracture	3 (2.3%)
Obstetric	
LSCS for Fetal distress/ scar rupture	57 (44%)
Oto-rhino-laryngology, Head and Neck surgery	
Bilateral FESS for Rhino-orbital mucor-mycosis	18 (14.7%)
Unilateral FESS for Rhino-orbital mucor-mycosis	21 (15.5%)
